# The Potential of Intralesional Rose Bengal to Stimulate T-Cell Mediated Anti-Tumor Responses

**DOI:** 10.4172/2155-9899.1000343

**Published:** 2015-07-22

**Authors:** Ajay V Maker, Bellur Prabhakar, Krunal Pardiwala

**Affiliations:** 1 Division of Surgical Oncology, Department of Surgery, University of Illinois at Chicago, Chicago Illinois, USA; 2 Department of Microbiology and Immunology, University of Illinois at Chicago, Chicago Illinois, USA

**Keywords:** Rose bengal, T-cell mediated anti-tumor responses, metastatic melanoma, PV10

## Abstract

Rose Bengal (RB) is a red synthetic dye that was initially used in the garment industry and has been used safely for decades as a corneal stain by ophthalmologists. Antineoplastic properties of RB have also been observed, though the mechanism of action remained to be elucidated. Recently, interest in RB as a therapeutic cancer treatment has increased due to significant anti-tumor responses with direct tumor injection in human clinical trials for metastatic melanoma. In these patients, there has been the implication that RB may mount a T-cell mediated anti-tumor response and impart antigen-specific responses in distant bystander lesions. This article serves to evaluate the potential of intralesional rose bengal to stimulate T-cell mediated anti-tumor responses in in-vitro, pre-clinical, and clinical studies.

## Introduction

Rose Bengal (RB, 4,5,6,7-tetrachloro-2,4,5,7-tetraiodofluorescein disolium salt) is a xanthene dye produced by combining halogens with fluorescein that was patented in 1882. It was used originally as a wool dye, but found an application as a measure for hepatic function and, in the early 1900’s, as a treatment for ocular pneumococcal infections [1-3]. This led to the discovery of its use as an effective stain for corneal ulcers; an application for which it is still used today [4-6]. It is a photosensitizer with well-characterized phototoxicity in living cells that results in the release of reactive oxygen species [7]. Using multiphoton microscopy, murine hepatoma cells treated with Rose Bengal and activated with green light demonstrated nearly immediate photolytic release of lysozymes that resulted in autolysis within 30 minutes [8,9]. RB appears to distribute within the cytoplasm, not the nucleus, and it is hypothesized that due to its anionic charge it has a proclivity for concentrating within lysosomes [8,9]. Importantly, the necrosis does not appear to denature tumor antigens. Melanoma cells treated in-vitro with RB induced light-independent cytotoxicity that was found to be dose-dependent and secondary to autophagy and cell necrosis [10]. These observations established a foundation on which to study its anti-tumor effects.

## Anti-tumor Cytotoxicity

In the 1980’s, RB was a commonly used red food dye in Japan. The effect of iodine containing RB on thyroid tumorigenesis ironically showed that mice treated with RB experienced longer survival and formed less tumors than control mice [11]. This fact was not fully appreciated until decades later when intralesional RB injection was found to result in tumor autolysis after photostimulation [8]. Thereafter, PV-10, a 10% formulation of RB that is not dependent on photostimulation for cytotoxic effects, was developed and is currently in trials for its anti-tumor efficacy.

### Melanoma

A phase I study with 11 patients had a total of 26 dermal metastatic lesions treated with intralesional injection of PV-10. At 12 weeks follow up, 19 lesions (76%) had a clinical response. Of these, nine experienced a complete response (CR), three a partial response (PR), and seven had stable disease. Interestingly, some untreated lesions in these patients also experienced a clinical response with a 15% CR, 12% PR, and with 31% displaying stable disease [12,13]. Promising results from this small sample prompted a multi-center, single-armed phase II trial. Eighty patients were enrolled and had up to 20 lesions treated. Some patients received 4 treatments every 4 weeks, while most patients received 1-2 treatments. These patients were followed for 52 weeks. Fifty-one percent of the patients experienced a clinical response with a 26% CR rate. When all or most of the known disease was treated with PV-10, greater response rates were seen when compared to patients in who significant disease was left untreated [12,14].

### Breast Cancer

Animal experiments have demonstrated that PV-10 may have antineoplastic activity against breast cancer. Balb/c mice were inoculated with MT-901 murine mammary carcinoma cells subcutaneously in 2 locations. When tumors were established, one of the tumors on each animal was injected with 50 μL of PV-10 or PBS. After intralesional injections, animals treated with PV-10 had statistically smaller treated (p<0.001) and untreated tumors (p<0.05) [15].

### Ovarian Cancer

In-vitro studies of the effect of RB on human ovarian cancer cells with and without BRCA1 mutation have been performed. When cultured with various concentrations of RB, both a BRCA1 mutation cell line, UWB, and wild-type BRCA1 expressing ovarian cancer cells showed dose-dependent growth suppression. Growth suppression of human fibroblasts was seen only at the highest concentration of RB, implying that lower dose RB had a selective effect on malignant cells [7]. This was consistent with prior reports that RB is known to be taken up in lysosomes of transformed cells, but not fibroblasts [8,16]. Colorimetric analysis for DNA fragmentation was completed in ovarian cancer cells treated with RB and compared to untreated cancer cells. Up to four-fold greater DNA fragmentation was detected in UWB cells treated with RB implying a greater degree of apoptosis occurring as a result of RB treatment. Further analysis of cell death revealed greater reactive oxygen species (ROS) generation. Cells treated with 50 mM RB produced similar levels of ROS as compared to 1000 mM of H_2_O_2_ [7].

### Gastric Cancer

The human gastric cancer cell line AGS was treated with RB in increasing concentrations. A dose-dependent suppression in cell proliferation was noted in AGS cells via MTT assay while a similar effect was not seen in a non-transformed fibroblast cell line. The mechanism of cell death was studied with Annexin V and PI staining and revealed a prevalence of apoptotic cell death as opposed to necrotic cell death [17].

### Sarcoma

In two patients with refractory scalp sarcoma – a leiomyosarcoma and a malignant fibrous histiocytoma (MFH) – tumors were treated with intralesional RB. The patient with leimyosarcoma had 9 lesions treated with RB and all lesions experienced a durable complete response at 5 months. The second patient had a single lesion of recurrent MFH that was treated successfully with RB. At 2 months, the patient presented with a recurrence, which was again treated with RB. The patient went on to develop pulmonary metastasis requiring systemic therapy [18].

### Summary of anti-tumor cytotoxicity

Rose Bengal is directly cytotoxic and has been found to induce lysozyme induced and apoptotic cell death in various human and murine malignancies when injected intralesionally. There is evidence that this effect may be specific to tumor cells. Some of these responses are durable, and treatment is successful in reducing overall tumor burden in selected patients. Ongoing clinical trials in melanoma may better define its role in this disease, though there is also potential in multiple malignancies based on in-vitro and pre-clinical studies. Of great interest is the effect intralesional treatment has been found to have incidentally on non-treated, or bystander, lesions. This nonrandom finding has piqued interest in the potential of RB induced cell death to generate a tumor-specific immune response or to expose tumor antigens for T-cell presentation.

## Induction of Anti-Tumor Immunity

In human clinical trials of melanoma patients with multiple subcutaneous metastases treated with intralesional RB, anti-tumor responses were observed in non-treated bystander lesions [12-14]. In 42 of 80 patients with designated bystander lesions, 26% experienced a CR in an uninjected bystander lesion, 7% a PR, and 17% demonstrated stable disease. Responses correlated with that of patients’ target lesions with a CR or PR in target disease corresponding with a 56% CR and 6% PR in bystander lesions. On the other hand, patients who did not experience an objective response of their target lesions experienced only a 6% CR and 12% PR rate (P=0.023) [14]. These findings may imply that tumor response in the primary lesion may be able to prime the immune system of patients for activity against distant lesions. Evaluation of peripheral blood samples on days 7-14 after RB treatment showed increased populations of T-cells and natural killer T-cells compared to pre-treatment, corresponding with increased interferon-gamma production from purified T-cells when exposed to autologous tumor, suggesting the possibility of antigen-specific T-cell activation and proliferation [19].

In a corresponding murine model with flank B16 melanoma tumors, the authors further demonstrated that tumor draining lymph nodes from RB treated tumors contained greater numbers of dendritic cells than non-draining lymph nodes, implying the possibility of greater antigen presenting cell activation and proliferation as a result of RB treatment. Additional experiments injected B16 murine melanoma cells in the flank and simultaneously via tail vein for the induction of lung metastases in C57B6 mice. Seven days later, flank tumors were injected with RB and on day 14 lungs were harvested. All 5 control injected animals demonstrated >250 lung metastases, whereas in 3/5 RB treated animals, <4 metastases were established [15]. Adoptive transfer of T-cells from PV-10 treated mice into sublethally irradiated B16-tumor bearing mice also slowed tumor growth in these animals and T-cells demonstrated increased levels of interferon-gamma (IFN-g) release.

Similarly, splenocytes from mice with PV-10 treated MT-901 murine breast cancer tumors were re-exposed to tumor cells in-vitro and IFN-g levels were assessed with ELISA. Almost 5 times greater IFN-g levels were observed in supernatants when splenocytes were collected from PV-10 treated animals. Increased INF-g production was specific to MT-901 cells as no difference was observed when the splenocytes were exposed to syngenic colorectal cancer cells [15]. These experiments established that in human and murine melanoma and breast cancer, there appeared to be an antigen-driven T-cell response that has the potential to activate T-cells and impart anti-tumor responses in bystander lesions and distant metastases.

Furthermore, in syngeneic orthotopic models of murine hepatocellular carcinoma (HCC), RB similarly induced chemoablation in all treated tumors. Twenty-one to 81 days after RB treatment, when re-challenged with the same tumor, durable immunity was demonstrated in 14/14 animals without measurable tumor formation, whereas B16-F10 melanoma tumors, i.e., non-HCC cells, were able to be established in 13/13 animals [20]. Additionally, immunity to establishment of a new HCC tumor could be created through adoptive transfer of splenocytes from treated animals. This was an interesting finding given that analysis of splenic composition from animals with B16 melanoma that experienced distant lung tumor regression after RB treatment of flank tumors did not demonstrate any difference in the percent of T cells, T_regs_, NK cells, B cells, myeloid derived suppressor cells, or macrophages [15]. The authors further demonstrated that bystander lesions disappeared or decreased in size in HCC models, whereas, no bystander tumors were ever observed to resolve in nude mice without a competent T-cell immune system. These experiments established that, similar to melanoma, in immunocompetent mice with orthotopic primary hepatocellular carcinoma flank tumors, an anti-tumor response can be induced by priming the animals with RB treatment of tumors. These experiments raise the possibility that RB induced cytotoxicity exposes antigens and mounts an immune response that may protects animals from additional tumor formation that can be adoptively transferred to other animals using splenocytes.

## Conclusion

Rose Bengal has been studied recently in several malignancies as an intralesional therapeutic agent. Experiments evaluating the mechanism of action demonstrate that RB is taken up selectively in the lysozomes of transformed cells [8,9,16]. It induces cell death by apoptosis and necrosis. Upon treatment with RB in-vivo, many treated patients and animals have been found to generate a systemic anti-tumor response resulting in growth suppression of untreated lesions. RB-mediated tumor cell death may expose tumor antigens that may otherwise evade immune detection. Upon re-exposure to these antigens, a robust cytotoxic T-cell response has been demonstrated in experimental models. Though most profoundly described in melanoma cells, clearly an immunoreactive malignancy, the effect has been shown to be not limited to one specific type of malignancy. Our current research is establishing the role of RB in generating anti-tumor immune responses in gastrointestinal cancer and liver metastases. Decrease in tumor burden and stimulation of an immune response with PV-10 has been demonstrated in animal models of metastasis, and correlations of these responses in clinical studies is consistent with such results. That PV-10 treatment can potentially increase circulating cytotoxic T-cells, even in patients who were previously treated with immune-activating checkpoint blockade, supports the possibility that RB induced cytotoxicity may activate T-cells that are responsible for the bystander effect on untreated lesions [19,21]. As such, intralesional therapy with RB may be a promising new mode of therapy to stimulate T-cell mediated anti-tumor immune responses.
